# Phytopharmaceutical practices of traditional health practitioners in Burkina Faso: a cross-sectional study

**DOI:** 10.1186/s12906-023-04055-z

**Published:** 2023-06-30

**Authors:** Kampadilemba Ouoba, Hélène Lehmann, Arsène Zongo, Rasmané Semdé, Jean-Yves Pabst

**Affiliations:** 1 Laboratory of Drug Development, Centre for Training, Research and Expertise in Drug Sciences, Doctoral School of Sciences and Health, Joseph Ki-Zerbo University, 03 BP 7021 Ouagadougou, Burkina Faso; 2 EA7307, Centre for International and European Studies, Faculty of Pharmacy, University of Strasbourg, 74, Route du Rhin, 67400 Illkirch, France; 3 EA4487, Centre for Research in Law and Legal Perspective, Faculty of Pharmacy, University of Lille, 3 Rue du Professeur-Laguesse, BP 53, 59006 Lille, France; 4 Faculty of Pharmacy, Université Laval, 1050 Avenue de La Médecine, Quebec City, QC Canada; 5 Axe Santé Des Populations Et Pratiques Optimales en Santé (SP-POS), Centre de Recherche du CHU de Quebec, 1050 Chemin Ste-Foy, Quebec City, QC Canada

**Keywords:** Traditional health practitioners, Phytopharmaceutical practices, Medicinal plants, Traditional medicines, Burkina Faso

## Abstract

**Background:**

Traditional health practitioners constitute an important part of the health care system in Burkina Faso, particularly in the supply of traditional herbal medicines. Quality and safety of these medicines rely heavily on practices employed during their traditional development. However, traditional phytopharmaceutical practices are poorly described in Burkina Faso. This study aimed to describe the phytopharmaceutical practices of traditional medicine practitioners in Burkina Faso.

**Methods:**

This was a cross-sectional descriptive ethno-pharmaceutical study, conducted from October 1 to November 30, 2020, among traditional practitioners in four randomly selected health districts: Nongr-Massom (Centre region), Tenkodogo (Centre-East region), Diapaga (East region) and Dafra (Hauts-Bassins region). An anonymous semi-structured face-to-face questionnaire was used to collect socio-demographic data and data on raw materials and finished products.

**Results:**

Sixty-seven (67) traditional health practitioners, aged 56 years on average, including a majority of men (72%), participated in the study. Gathering of wild medicinal plants was the main source of raw materials acquisition (51.5%), which were usually leaves (32.3%). These raw materials were usually sun-dried (43.9%) and packaged mostly in plastic bags (37.2%). They were derived from 60 plant species belonging to 33 botanical families. *Fabaceae* was the most represented family (18.7%) and *Khaya senegalensis* Juss. (*Meliaceae*) the most cited plant species (5.2%). The finished products had an average shelf life of 17 months and were usually prepared as a decoction (31.7%) and administered most often orally (71.4%). Gastrointestinal disorders were the main predictable adverse events of the finished products (54%).

**Conclusion:**

This study showed that THPs have important knowledge in the use of medicinal plants, but several shortcomings are observed in their phytopharmaceutical and plant protection practices. Continuous improvement of these practices, through education and training of traditional health practitioners, is essential for the conservation of plant biodiversity and quality assurance of traditional herbal medicines.

## Background

For ages, traditional medicine (TM) has been a health care system accessible to millions of people in Africa [1, 2]. Despite the emergence of so-called conventional therapies, TM remains the main health care for a significant proportion of the population in Africa, due in particular to the inaccessibility of modern health care and pharmaceutical products. It also pertain to some perceived safety of remedies of natural origin and their good cultural acceptability [2–6].

Traditional healers, key actors in the traditional health care system, ensure the continuity and transmission of endogenous medical knowledge from generation to generation in different forms, the main one being oral parental transmission [2, 7]. The imparted knowledge focuses on traditional medical material, the source of the traditional pharmacopoeia. This pharmacopoeia is defined as the body of knowledge, preparation techniques and use of natural plant, animal and/or mineral substances, which serve to diagnose, prevent or eliminate an imbalance in physical, mental or social well-being [8]. Studies in several African countries have shown that apart from their role in providing health care to the population, TM practitioners are also involved in safeguarding biodiversity, thus contributing to sustainable development [1, 9–11].

In the case of Burkina Faso, a large proportion of the population, especially in rural areas, uses medicinal plants and TM to treat common illnesses such as malaria and febrile childhood illnesses [12]. This landlocked West African country is characterized by a low proportion of the population having access to public health care and a ratio of allopathic doctors to patients of about 1 per 20,000 [13]. It covers an area of 274,200 km^2^, with more than 20 million inhabitants, the majority of whom (69.4%) live in rural areas [14]. More than 40% of the population lives below the poverty line and the country has more than 60 ethnic groups, the main ones being the Mossi (more than 48%), the Fulani (more than 10%), the Bobo (more than 7%), the Gulmantchéba and the Gurunsi (each more than 7%) [14]. Biogeographically, the country extends from the Sahelian zone in the north to the Sudanian zone in the south and is located between the sub-arid and sub-humid zones [15, 16]. The main soil types found in Burkina Faso are tropical ferruginous [17]. Floristically, the country is subdivided into four phytogeographic zones – Southern Sudanian, Northern Sudanian, Sub-Sahelian and Sahelian [18]. The average annual rainfall increases from the Sahelian zone (400 mm) to the Sudanian zone (1000 mm), while the average annual temperature decreases [18]. The vegetation belongs to the regional Sudanese endemic centre and the Sahelian transition zone [18]. It is dominated by steppes in the north, savannahs in the centre and south and a few patches of dry forest in the south of the country. The flora is typically Sahelo-Sudanese and estimated at 2067 plant species [19]. In epidemiological terms, the country's health profile is strongly marked by endemic diseases such as malaria and dengue fever, and by infectious childhood diseases [4, 13].

Thus, this floristic diversity combined with the inadequate supply of allopathic medicine, poverty and deeply rooted cultural practices, makes TM a crucial and legally recognized element of the population's health care in Burkina Faso since 1994 by the public health code [20]. The current regulation on TM in Burkina relates to the authorization to practice TM, the authorization to open traditional health establishments, the registration process of herbal TMs and the collaboration system between conventional medicine and TM [21]. Burkina Faso’s TM is therefore practiced by traditional health practitioners (THPs) who produce traditional medicines (TMs) available to rural and urban populations for their priority health care needs [4, 20]. In 2020, the annual prevalence of TMs use by the general population was 85%, including 54.3% acquiring the medicinal products exclusively from THPs [4]. In addition, only six TMs manufactured in Burkina Faso obtained the country marketing authorizations (MA) in 2020, although the registration of medicines from the traditional pharmacopoeia is subject to a lighter procedure, compared to pharmaceuticals [22]. Among other reasons for this low rate of TMs registration, meeting the quality, efficacy and safety requirements, even for medicines derived from the traditional African pharmacopoeia constitute a hurdle often too high for traditional practitioners [23]. At the national level, several ethnobotanical studies for the identification of medicinal plants for fundamental research and those relating to the use of TMs in the general population have been carried out [4, 12]. However, no study has previously looked at the phytopharmaceutical practices of traditional health practitioners, who are the repositories of traditional medical knowledge. Yet, these practices form the basis for the TMs' quality assurance system, the foundation for their integration into the formal health system in Burkina Faso [24–26].

The objective of this pioneering work was to analyze and document the traditional phytopharmaceutical practices of the THPs in Burkina Faso, in particular by describing the procurement and processing of raw materials, the methods of preparation and use of finished products, and expected adverse events (AEs).

## Methods

### Setting, type and period of the study

**Fig. 1:**
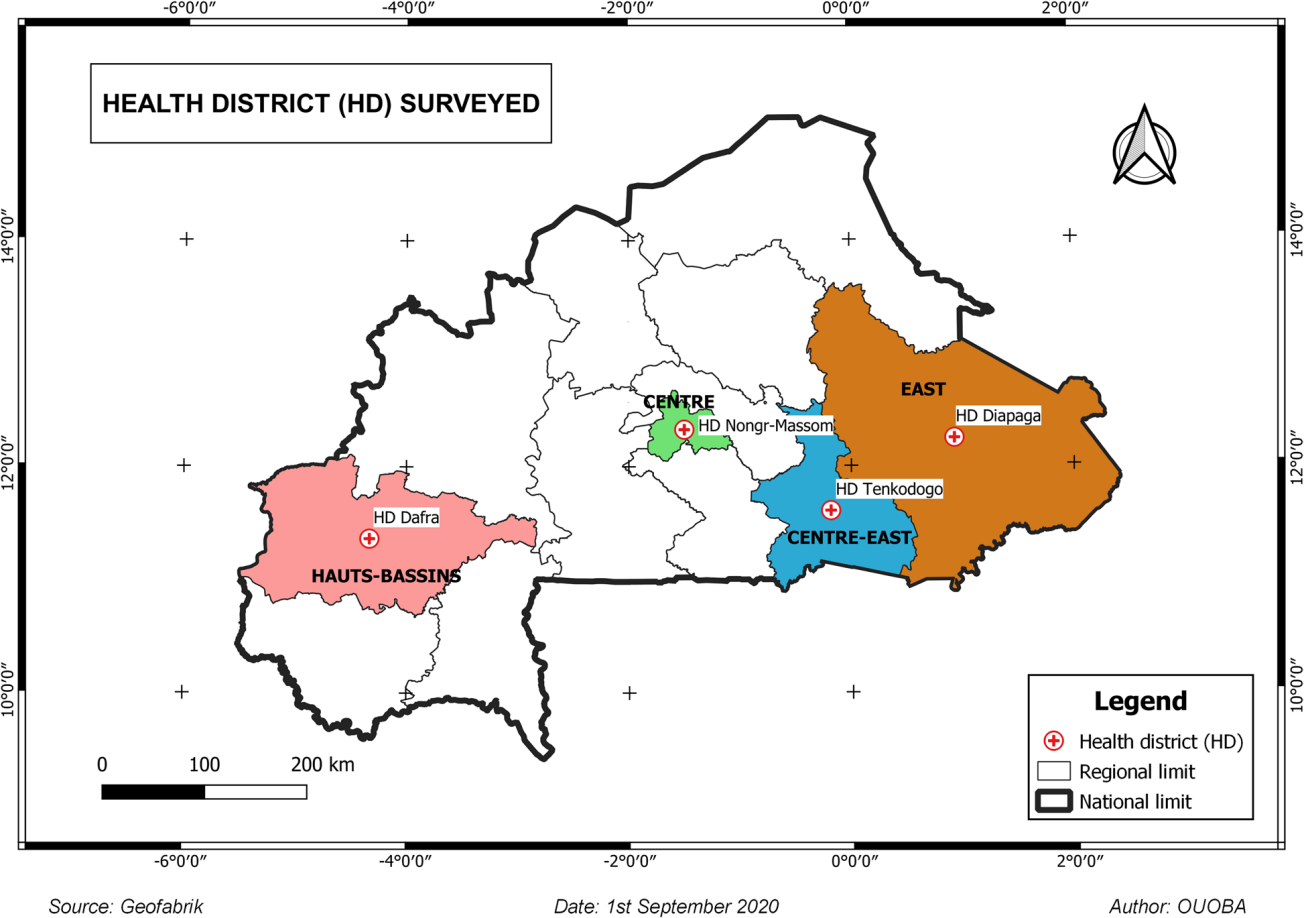
Regions and health districts surveyed

A cross-sectional, descriptive ethno-pharmaceutical study was conducted from October 1 to November 30, 2020, in four health regions of the country, namely Centre region, Centre-East region, East region and Hauts-Bassins region. These four regions were selected at random from the thirteen regions of Burkina Faso. For each selected health region, a health district (HD) was randomly designated for the survey, namely the HDs of Nongr-Massom (Centre region), Tenkodogo (Centre-East region), Diapaga (East region) and Dafra (Hauts-Bassins region) (Fig. 1).

### Population sampling and inclusion criteria

The study involved THPs aged 20 years or older who were practicing TM in the health areas of the selected districts. The directories of THPs in the selected HDs were used for the selection of THPs in the study, on the conditionality of their consent. All THPs whose identities, telephone contacts and membership in a legally recognized association of THPs were listed. THPs who could not be reached through their telephone number during the study period were not included. Thus, a total number of 190 THPs was listed in the four selected HDs: 87 (HD Nongr-Massom), 32 (HD Tenkodogo), 19 (HD Diapaga) and 52 (HD Dafra). Of these, 68 could not be reached during the study period and 55 others did not consent to participate in the study, bringing the number of THPs interviewed to 67.

### Data collection technique and tool

A semi-structured questionnaire was used to collect informations. The questionnaire was administered to THPs face-to-face in their native language by trained and qualified interviewers.

The questionnaire was divided in three parts:

- Part 1 – sociodemographic and professional data of the participants: age, gender, marital status, educational level, religion, area of residence, professional category, source of acquisition of traditional medical knowledge, diseases treated by THPs, initial and continuing education;
- Part 2 – raw materials and finished products: origin, methods of drying, methods of preservation and packaging of raw materials; local or vernacular names of source plants/animals/minerals; methods of preparation, packaging and preservation of finished products;
- Part 3 – instructions for use and safety of finished products: dosage regimen, contraindications, expected AEs and effects of overdose.

### Ethical considerations.

The study protocol was approved by the National Ethics Committee for health research of Burkina Faso, n°2020–9-201 on 2 September 2020.

An information sheet was made available to study participants. It included information about the title, objectives, study setting, research team, voluntary participation in the study, anonymity of the questionnaire by assigning an anonymous numerical code, confidentiality of data, the course of the survey and telephone contacts of the principal investigator and the National Ethics Committee.

THPs were interviewed after obtaining their informed signatures on a pre-determined form. A witness signature was requested when the THPs could not read or write. The confidentiality of personal data and the anonymity of the participants were guaranteed during the data collection and processing.

### Data capture and analysis

The data were entered using Sphinx version 5 software and then analyzed using Statistical Package for Social Science (SPSS) version 25 software. Descriptive statistics—numbers, frequencies, means, standard deviations, medians—were used to process the results. The names of the medicinal plants cited by the THPs were collected in their common names in French or in the main local languages of the country, notably Mooré and Dioula. The Latin binomials and families of plant species were then obtained using the national guide to medicinal plants in Burkina Faso, and then verified on www.theplantlist.org and www.worldfloraonline.org [27, 28].

## Results

### Sociodemographic and professional characteristics

Men were more represented (72%), with a sex ratio of 2.5. The average age of the participants was 56 ± 12 years. THPs with education up to primary level represented the highest number of study subjects (83.6%). The majority of THPs were Muslim (57%) and most resided in urban areas (78%). Most of the THPs surveyed had more than 20 years of work experience (58.2%). Gastrointestinal diseases (46.2%), malaria (25.4%), infectious diseases (22.4%), sinusitis (16.4%) and viral hepatitis (13.4%) were the main diseases treated by THPs. The main source of acquisition of traditional medical knowledge was oral transmission (51%). More than half of the THPs had not received training in either good agricultural and collection practices for medicinal plants (59%), good manufacturing practices for TMs (56%), or TMs safety (54%) (Table 1).

**Table 1:** Sociodemographic and professional characteristics of participants (*n* = 67)

Variables	Frequency, n (%)
**Number of THPs**^a^	
Nongr-Massom	17 (25.4)
Dafra	17 (25.4)
Tenkodogo	15 (22.2)
Diapaga	18 (27)
**Age (years)**	
< 55	27 (40)
≥ 55	40 (60)
**Marital status**	
Married	57 (85.1)
Single	2 (3)
Divorced	1 (1.5)
Widow (er)	7 (10.4)
**Gender**	
Male	48 (72)
Female	19 (28)
**Education level**	
None	29 (43.3)
Primary	27 (40.3)
Secondary	9 (13.4)
University	2 (3)
**Religion**	
Animist	12 (18)
Christian	17 (25)
Muslim	38 (57)
**Residence area**	
Urban	52 (78)
Rural	15 (22)
**Professional seniority (years)**	
0—5	3 (4.5)
6—10	9 (13.4)
11—15	5 (7.5)
16—20	11 (16.4)
> 20	39 (58.2)
**Main diseases treated by THPs**	
Gastrointestinal diseases	31 (46.2)
Malaria	17 (25.4)
Infectious diseases	15 (22.4)
Sinusitis	11 (16.4)
Viral hepatitis	9 (13.4)
Asthma	8 (12)
Infertility	7 (10.3)
Rheumatism	6 (9)
High blood pressure	6 (9)
Diabetes	4 (6)
**Source of traditional medical knowledge (*n* = 98)**	
Oral parental transmission	50 (51)
Learning from another THP	21 (21.4)
Written parental transmission	9 (9.2)
Initial or professional training in TM^b^	9 (9.2)
Through revelation	9 (9.2)
**Already received training on good agricultural and collection practices for medicinal plants (*n* = 64)**	
No	38 (59)
Yes	26 (41)
**Already received training on good manufacturing practices for TMs**^**c**^** (*n* = 64)**	
No	36 (56)
Yes	28 (44)
**Already received training on TMs safety (*n* = 65)**	
No	35 (54)
Yes	30 (46)

^a^*THPs * Traditional health practitioners,  ^b^*TM*  Traditional medicine, ^c^*TMs*  Traditional medicines

### Raw materials and finished products

More than half of the THPs (51.5%) obtained their raw materials by gathering them from plants or animals. They were mostly plant parts (91.8%) and usually dried in the sun (43.9%) or in the shade (42.5%), in the open air on tarpaulins, then packaged mostly in plastic bags (37%). The finished products were also often packaged in plastic bags (58%) and had to be prepared as a decoction (31.7%) or infusion (22.2%). The majority of these products (64.2%) were labelled with at least the instructions for use, shelf life, identity and address of the manufacturer. The products were generally stored at room temperature in a dedicated clean room (76.5%), with an average shelf life of 17 months. The majority of products (55.4%) had a shelf life of between one and two years (Table 2).

**Table 2:** Data on raw materials and finished products

Variables	Frequency, n (%)
**Sources of raw material supply (*n* = 99)**	
Gathering in the flora or fauna	51 (51.5)
Buying from a medicinal plant wholesaler	38 (38.4)
Growing medicinal plants	10 (10.1)
**Natural origins of raw materials (*n* = 135)**	
Plant	124 (91.8)
Animal	9 (6.7)
Mineral	2 (1.5)
**Raw material drying methods (*n* = 73)**	
Sun drying in the open air on tarpaulins	32 (43.9)
Shade drying in the open air on tarpaulins	31 (42.5)
Shade drying on the ground in the open air	4 (5.5)
Sun drying in the open air on the ground	2 (2.7)
Sun drying then shade drying in the open air on tarpaulins	2 (2.7)
Shade drying in an enclosed area on tarpaulins	2 (2.7)
**Primary packaging of raw materials (*n* = 43)**	
Plastic bag	16 (37)
Plastic drum	8 (19)
Cardboard	6 (14)
Clay pot	6 (14)
Plastic bottle	4 (9)
Piece of fabric	3 (7)
**Preparation methods for finished products (*n* = 126)**	
Decoction	40 (31.7)
Infusion	28 (22.2)
Calcination/Carbonisation	21 (16.7)
Powder	21 (16.7)
Maceration	16 (12.7)
**Primary packaging of finished products (*n* = 64)**	
Plastic bag	37 (58)
Plastic bottle	10 (15.5)
Plastic drum	5 (8)
Glass bottle	3 (4.5)
Piece of fabric	3 (5)
Calabash	2 (3)
Cardboard	2 (3)
Paper	2 (3)
**Labelling of finished products with instructions for use, identity and address of manufacturer (*n* = 95)**	
No	34 (35.8)
Yes	61 (64.2)
**Storage conditions for finished products (*n* = 17)**	
Suitable location at room temperature	13 (76.5)
Suitable room free of moisture	3 (17.5)
Anywhere in the home	1 (6)
**Registration of finished products (*n* = 96)**	
No	91 (94.8)
Yes	5 (5.2)
**Shelf life of finished products (years) (*n* = 74)**	
< 1	20 (27)
1 – 2	41 (55.4)
3 – 4	12 (16.2)
> 4	1 (1.4)

**Fig. 2:**
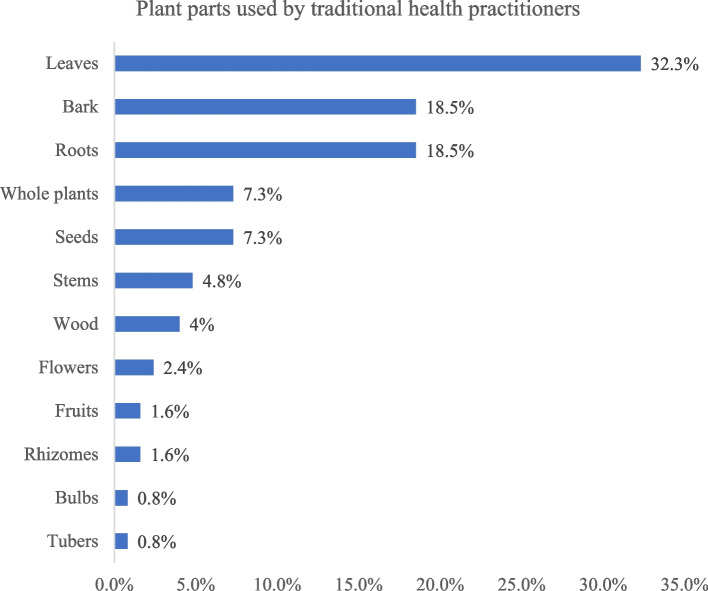
Plant parts used by traditional health practitioners

The plant materials exploited by the THPs were mainly leaves (32.3%), bark (18.5%) and roots (18.5%) (Fig. 2). They were derived from 60 plant species belonging to 33 botanical families, of which *Fabaceae* (18.7%), *Poaceae* (8.4%) and *Meliaceae* (8.4%) were the most cited. *Khaya senegalensis* Juss. (*Meliaceae*) was the most mentioned plant species (5.2%) (Table 3).

**Table 3:** Medicinal plants cited by traditional health practitioners (total number of citations, *n* = 95)

Botanical family	Plant species	Local name (French, Dioula^a^ or Mooré^b^)	Frequency species, n (%)	Family frequency, n (%)
*Alliaceae*	*Allium cepa* L	Oignon	1 (1)	1 (1)
*Amaranthaceae*	*Achyrantes aspera* L	Herbe d’Eugène ou Baag-yoré (Mooré)	1 (1)	1 (1)
*Amaryllidaceae*	*Crinum zeylanicum* (L.) L	Yeemdé (Mooré)	2 (2.1)	2 (2.1)
*Anacardiaceae*	*Lannea microcarpa* Engl. & K. Krause	Raisinier africain ou Sabga (Mooré)	1 (1)	
	*Mangifera indica* L	Manguier	1 (1)	4 (4)
	*Lannea acida* A. Rich	Raisinier acide, Sabtulga (Mooré)	1 (1)	
	*Lannea velutina* A. Rich	Waamsabga (Mooré)	1 (1)	
*Annonaceae*	*Polyalthia longifolia* (Sonn.) Thwaites	Arbre mat ou Faux ashoka	2 (2.1)	2 (2.1)
*Apocynaceae*	*Alstonia boonei* De Wild	Bois de tabouret	2 (2.1)	3 (3.1)
	*Leptadenia pyrotechnica* (Forssk.) Decne	Sarafato (Dioula)	1 (1)	
*Bombacaceae*	*Bombax costatum* Pellegr. & Vuillet	Kapokier à fleurs rouges, Voaka (Mooré)	1 (1)	1 (1)
*Brassicaceae*	*Brassica rapa* L	Yolga (Mooré)	1 (1)	1 (1)
*Caricaceae*	*Carica papaya* L	Papayer	2 (2.1)	2 (2.1)
*Cochlospermaceae*	*Cochlospermum planchonii* Hook.f	N’dribala (Dioula)	2 (2.1)	2 (2.1)
*Combretaceae*	*Combretum paniculatum* Vent	Combrétum paniculé	2 (2.1)	3 (3.1)
	*Pteleopsis suberosa* Engl. & Diels	Guirga (Mooré)	1 (1)	
*Compositae*	*Acanthospermum hispidum* DC	Suraka voni (Dioula)	1 (1)	1 (1)
*Convolvulaceae*	*Ipomea batatas* (L.) Lamb	Patate douce	1 (1)	1 (1)
*Ebenaceae*	*Diospyros mespiliformis* Hochst. ex A. DC	Ebénier de l’Ouest africain ou Gaaka (Mooré)	1 (1)	1 (1)
*Euphorbiaceae*	*Chrozophora brocchiana* (Vis.) Schweinf	Bund-yaaba (Mooré)	1 (1)	4 (4)
	*Alchornea cordifolia* (Schumach. & Thonn.) Müll.Arg	Arbre de djeman, Djeka (Dioula)	1 (1)	
	*Euphorbia pulcherrima* Willd. ex Klotzsch	Manioc rouge	1 (1)	
	*Jatropha multifida* L	Arbre au corail, Wabinbanguema (Mooré)	1 (1)	
*Fabaceae*	*Cassia alata* L	Jonis tiiga	2 (2.1)	18 (18.7)
	*Trigonella foenum-graecum* L	Fenugrec	1 (1)	
	*Caesalpinia pulcherrima* (L.) Sw	Orgueil de chine	2 (2.1)	
	*Afzelia africana* Smith ex Pers	Haricot acajou ou Kankalga (Mooré)	1 (1)	
	*Tamarindus indica* L	Tamarinier	2 (2.1)	
	*Acacia nilotica* (L.) Willd. ex Delile	Acacia à gommier rouge ou Bagana (Dioula)	1 (1)	
	*Acacia polyacantha* Willd	Catéchu africain	2 (2.1)	
	*Cassia occidentalis* L	Kinkéliba	2 (2.1)	
	*Arachis hypogaea* L	Arachide	1 (1)	
	*Parkia biglobosa* (Jacq.) G. Don	Néré	3 (3.2)	
	*Canavalia ensiformis* (L.) DC	Œil de cheval, Peraogo (Mooré)	1 (1)	
*Lamiaceae*	*Tinnea barteri* Gürke	Kinkirs kaana (Mooré)	3 (3.2)	3 (3.2)
*Loranthaceae*	*Agelanthus dodoneifolius* (DC.) Polhill & Wiens	Gui africain ou Welba (Mooré)	3 (3.2)	3 (3.2)
*Malvaceae*	*Adansonia digitata* L	Baobab ou Pain de singe	2 (2.1)	5 (5.1)
	*Gossypium Ssp.*	Cotonnier	1 (1)	
	*Ceiba pentandra* (L.) Gaertn	Faux kapokier ou Gounga (Mooré)	1 (1)	
	*Cienfuegosia digitata* Cav	Salguende (Mooré)	1 (1)	
*Meliaceae*	*Khaya senegalensis* Juss	Caïlcédrat	5 (5.2)	8 (8.4)
	*Azadirachta indica* A. Juss	Neem	3 (3.2)	
*Monimiaceae*	*Peumus boldus* Molina	Boldo	1 (1)	1 (1)
*Myrtaceae*	*Eucalyptus globulus* Labill	Eucalyptus	3 (3.2)	3 (3.2)
*Olacaceae*	*Ximenia americana* L	Citronnier de mer ou Leenga (Mooré)	1 (1)	1 (1)
*Phyllanthaceae*	*Phyllanthus amarus* Schumach. & Thônn	Petit tamarinier blanc, Woom pooré (Mooré)	1 (1)	1 (1)
*Poaceae*	*Cymbopogon citratus* Stapf	Citronnelle	3 (3.2)	8 (8.4)
	*Zea mays* L	Maïs	3 (3.2)	
	*Sorghum bicolor* (L.) Moench	Sorgho, grand mil	1 (1)	
	*Pennisetum americanum* (L.) Leeke	Petit mil	1 (1)	
*Polygalaceae*	*Securidaca longipedunculata* Fresen	Pelga (Mooré)	1 (1)	1 (1)
	*Polygala amara* L	Polygale amer	1 (1)	1 (1)
*Rosaceae*	*Rubus idaeus* L	Flamboisier	1 (1)	1 (1)
*Rubiaceae*	*Spermacoce verticillata* L	Faux ipeca ou Yoadga (Mooré)	1 (1)	3 (3)
	*Gardenia ternifolia* Schumach. & Thônn	Lambre zunga (Mooré)	1 (1)	
	*Zanthoxylum zanthoxyloides* (Lam.) Zepern. & Timler	Rapeoko (Mooré)	1 (1)	
*Rutaceae*	*Citrus aurantiifolia* (Christm.) Swingle	Citronnier	3 (3.2)	3 (3.2)
*Sapotaceae*	*Vitellaria paradoxa* C.F. Gaertn	Karité	2 (2.1)	2 (2.1)
*Verbenaceae*	*Stachytarpheta indica* (L.) Vahl	Verveine ou queue de rat	1 (1)	1 (1)
*Zingiberaceae*	*Kaempferia galanga* L	Gingembre	3 (3.2)	3 (3.2)

^a^Dioula: fourth national language in Burkina Faso; ^b^Mooré: first national language in Burkina Faso

### Use and safety of finished products

The finished products were generally administered orally (71.4%). More than 60% of the products prescribed by the THPs with a recommended dosage. Age and sex of patients were taken in consideration for almost half of the products (49%). The duration of treatment was a maximum of five days for the majority of products (44.2%), in two daily doses, with an average treatment duration of 12 days. A table spoon (32.6%), glass (25%) or a finger pinch (19.6%) were the most common dosing materials. According to THPs, most of the products they held (62%) were not contraindicated for pregnant or breast-feeding women. Gastrointestinal disorders were both the main predictable AEs of the products (54%) and the subsequent toxic effects of their overdose (60.5%) (Table 4).

**Table 4:** Data on use and safety of finished products

Variables	Frequency, n (%)
**Routes of administration (*n***^**a**^** = 133)**	
Oral	95 (71.4)
Topical dermal	19 (14.2)
Inhalation	9 (6.8)
Bath	5 (3.8)
Rectal	3 (2.3)
Auricular	2 (1.5)
**Existence of dosage (*n* = 123)**	
No	44 (35.8)
Yes	79 (64.2)
**Age and gender dependent dosage (*n* = 121)**	
No	62 (51.2)
Yes	59 (48.8)
**Number of daily intakes (*n* = 129)**	
1	36 (27.9)
2	57 (44.2)
3	34 (26.4)
> 3	2 (1.6)
**Material or mode of dosing (*n* = 92)**	
Tablespoon	30 (32.6)
Glass	23 (25)
Pinch	18 (19.6)
Coffee spoon	5 (5.4)
Calabash	3 (3.3)
Handle	3 (3.3)
Capsule	2 (2.2)
Cup	2 (2.2)
Sip	2 (2.2)
Measuring bottle	1 (1.1)
Soaked cotton	1 (1.1)
Drop	1 (1.1)
Rectal enema pear	1 (1.1)
**Treatment duration (days) (*n* = 111)**	
≤ 5	49 (44.2)
6 – 10	26 (23.4)
11 – 15	10 (9)
> 15	26 (23.4)
**Can be used during pregnancy or breastfeeding (*n* = 121)**	
No	46 (38)
Yes	75 (62)
**Expected adverse events (*n* = 28)**	
Gastrointestinal disorders (diarrhea, vomiting, abdominal pain, nausea, burping)	15 (54)
Dizziness	3 (11)
Muscle pain	2 (7)
Drowsiness	2 (7)
Skin flaking	2 (7)
Large diuresis	2 (7)
Headache	1 (3.5)
Temporary giddiness	1 (3.5)
**Effects of overdose (*n* = 38)**	
Gastrointestinal disorders (diarrhoea, vomiting, bloating, constipation, nausea)	23 (60.5)
Vertigo	3 (8)
Hypersudation	2 (5.3)
Body pain	2 (5.3)
Palpitation	1 (2.6)
Headache	1 (2.6)
Heavy diuresis	1 (2.6)
Sneezing	1 (2.6)
Fever	1 (2.6)
Limbs inflammation	1 (2.6)
Irritation of the tongue	1 (2.6)
Discomfort	1 (2.6)

^a^*n* = number of products

## Discussion

The objective of this study was to describe the traditional phytopharmaceutical practices of traditional practitioners in Burkina Faso. The relatively low participation rate of the study (67 out of 190 listed) reveals certain difficulties in conducting studies among TM practitioners in Burkina Faso. The first concerns the absence of an updated national directory of THPs, making it difficult to sample this population during epidemiological studies. The second concerns the absence of a map of the places of practice of the THPs and the updating of their directories at community level. However, the current public health code provides in Article 143 that the organization of THPs shall be determined by regulation [20]. This will provide an up-to-date national database of THPs that will facilitate ethnomedical surveys. The final difficulty is the reluctance of some THPs to collaborate with biomedical practitioners or researchers. In this regard, Oseni et al. recently highlighted, from a qualitative systematic review, limitations in collaboration between indigenous and allopathic health practitioners in Africa, marked by lack of mutual understanding, rivalry, mistrust and lack of respect [29]. The reluctance of TM practitioners to collaborate with biomedical researchers was similarly observed in South Africa and Kenya, in 2020 [30, 31]. According to van Rooyen et al., these collaborative constraints could be overcome by facilitating mutual understanding through open communication between the two types of practitioners, based on mutual respect and acceptance [32].

The study showed that TM practitioners contribute to health care provision for the Burkinabè population. In fact, although the study covered only 67 THPs because of the inclusion criteria, it should be noted that a total of 190 THPs were listed in the health areas of the four districts surveyed and home to a total of 1,478,268 inhabitants, i.e. a ratio of 1.3 THPs/10,000 inhabitants [13]. At the same time, this ratio was 4.3 times higher than that of modern physicians (0.3 physician/10,000 inhabitants), with 52 physicians for the four HDs [13]. This demonstrates the major contribution of THPs to access to health care in Burkina Faso. In addition, the diseases treated by the THPs, which were mainly gastrointestinal diseases (46.2%), malaria (25.4%) and infectious diseases (22.4%), fit in line with the health profile of the areas surveyed and that of the rest of the country, which is dominated by gastrointestinal diseases, malaria and infectious diseases in children [12, 13]. In other African countries, studies have shown that traditional therapists represent a considerable health care resource, particularly in the management of primary diseases such as malaria, tuberculosis and HIV/AIDS [11, 33, 34].

Regarding the raw materials used by THPs, the results showed that most of them were plant parts (91.8%) and that wild plant collection remained the main source of supply (51.5%), with little agricultural growing of medicinal plants (10%). Herbal medicine is indeed the main therapy in traditional and complementary medicine, according to the WHO Global Report 2019 [35]. A previous study had shown that in Burkina Faso, the majority of traditional remedies used by the population were plant-based (93.5%) and were obtained mainly from THPs (54.3%) [4]. The same observation was reported in Ethiopia where traditional healers obtained their medicines mainly from natural plant substances for the basic health needs of the population [36].

The growing of medicinal plants was not a common practice in our study (10%). More than a decade ago (2011), an ethnobotanical study conducted in the Centre-East of Burkina Faso had already made this finding, indicating that wild plants were the main source of traditional healers' remedies and that the cultivation of medicinal plants was uncommon [12]. This was also found in Ghana where most of the plant materials (55%) used by traditional therapists were harvested from the bush [37]. This weakness would be due to the limitations of traditional reduced-scale irrigation methods for vegetables, which are commonly practiced manually in low-rainfall areas for home gardens. The compliance of these irrigation methods with medicinal plants would not allow a satisfactory profitability to sustain the activity [38]. An experiment in Niger reported that low-pressure drip irrigation can be an alternative to traditional irrigation methods in sub-Saharan Africa [38]. This improved irrigation method has shown a strong positive impact in the growing of medicinal plants, which can contribute to the sustainable development of African TM [38]. In addition to these structural constraints, a survey of South African traditional healers noted the existence of cultural hurdles to the growing of medicinal plants. The study found that several plant species were barred from cultivation due to cultural norms and ancestral guidelines [39].

Since TM is strongly associated with its socio-cultural acceptability, it is important to involve traditional medical knowledge holders in medicinal plant cultivation initiatives to ensure that these interventions are culturally acceptable to the beneficiaries [39]. Unsustainable exploitation of medicinal plant species, particularly in Sahelian countries, is a real threat to biodiversity [40–42]. Furthermore, it should be noted that the cultivation and harvesting of medicinal plants requires a national policy that includes strengthening the knowledge and skills of THPs in this area [25]. In our case, more than half of the THPs reported that they had never received training on good agricultural and collection practices for medicinal plants. Yet, the standardized production and cultivation of medicinal plants will be the starting point of the industrial chain of herbal TMs and can play a decisive role in the sustainable economic development of African TMs, just like in China [43]. This will not only safeguard plant biodiversity but also increase and sustain TM's contribution to achieving "health for all" [10, 44].

Regarding the medicinal plant material used by the THPs, it was mainly leaves (32.3%), bark (18.5%) and roots (18.5%). The same trend was observed in the ethnobotanical study conducted in 2011 in the Central East of the country, which reported that leaves (38.4%), barks (30%) and roots (29.5%) were the plant parts that are mostly used by the THPs, thus showing their consistent use of plant material for health care [12]. In some of Burkina Faso's neighboring countries such as Benin and Ghana, and even in further lands such as India, leaves are the most common plant material used by traditional healers [45–47].

The plant raw materials were derived from 60 plant species belonging to 33 botanical families of which the main ones were *Fabaceae* (18.7%), *Poaceae* (8.4%) and *Meliaceae* (8.4%) while *Khaya senegalensis* Juss. (*Meliaceae*) was the most cited plant species (5.2%). They were mainly used to treat gastrointestinal diseases and malaria, which are among the main reasons for consultations in health care facilities in Burkina Faso [48]. In Angola, the *Fabaceae* is also the botanical family most used by traditional therapists, but it is seconded by the *Phyllanthaceae*, unlike our case [49]. Climatic differences are thought to play a key role in plant diversity between countries or regions.

As regards the drying and packaging of raw materials, they were mostly dried in the sun (43.9%) or in the shades (42.5%), in the open air on tarpaulins; then packaged most often in plastic bags (37.2%). Although these practices are recognized by the WHO's good practices in this area, their effectiveness depends on the exact compliance with the conditions of use, in particular, temperature, humidity, drying time and packaging material [25]. Where medicinal plant material is to be used in a dry state, the water content should be minimized to prevent the growth of molds and other microbial agents [25]. It is recognized that the drying methods of plant materials play an essential role in the quality and safety of herbal medicines [25, 50]. They can affect the composition and physicochemical characteristics of many of the active chemical constituents, as well as the microbiological quality of finished products [25]. Thus, sun or shade drying depends on the nature of the active components of interest and the plant parts to be dried [25]. It has been shown in the literature that the essential oil content and composition of fragrant verbena (*Lippia citriodora* Kunth) varies considerably depending on the drying method – shade drying, freeze drying, oven drying or vacuum drying [51]. With regard to shade drying, it has been shown that it leads to a loss of the active component (bacoside A) of the memory-enhancing medicinal herb *Brahmi* (*Bacopa monnieri* L.) at the lowest level compared to other drying methods (sun, sun tunnel, cabinet) [50]. A minority of THPs (8.2%) air-dried on the floor, which is strongly discouraged due to the exposure to environmental pollutants associated with this practice [25].

Similarly, the packaging materials of dry raw materials also have a role in preserving their quality. In the case of *Bacopa monnieri* L., high density polyethylene packaging significantly reduces the loss of bacoside A compared to other packaging, regardless of the compliance instructions applied, and ensures better microbiological quality [50]. Another study indicated that the antibacterial and anti-inflammatory activity of some African medicinal plants changes as a result of packaging and depends on the nature of the packaging, the plant species and the temperature [52].

Regarding the finished products, they were mostly stored in appropriate premises at room temperature (76.5%), usually for one to two years (55.4%) and prepared mostly as a decoction (31.7%) for use. This would be based exclusively on the empirical experience of THPs and is in accordance with the nature of the plant parts used by the THPs, which were mostly leaves and could be stored for the recommended period when properly dried and packaged. Their preparation as a decoction is also easy to use. The population-based study in Burkina Faso reported that decoction was the main instruction for TMs (59.2%), followed by infusion (15.4%). This instruction for the preparation of TMs justifies their frequent oral administration, as indicated in the study (71.4%). A study on the ethno-pharmacological use of herbal remedies for the treatment of malaria conducted in Ghana also showed that most of these remedies were prepared by boiling and administered orally [46].

The majority of THPs claimed to have mastered the dosages of their medicines (64.2%), recommending two daily doses for up to five days for most of the medicines (44.2%), with the table spoon as the main dosing device (32.6%). These instructions, which are exclusively empirical, are based on the long experience of using these medicines, which is the only basis for their safety. Beyond the long experience of using these products, which does not solidly guarantee the safety and proper use of herbal TMs, efforts must be made in a collegial manner between health authorities, research centers and THPs, with a view to a minimum standardization of the modes of dosage and use of the herbal TMs most commonly used by the population, such as antimalarial TMs.

As for the safety of TMs, the majority of the finished products could be used in pregnant or breastfeeding women (62%), based on empirical knowledge about these products. This represents a potential risk due to the lack of factual information on the safety of their use in these at-risk individuals. Several studies have shown that herbal medicines or herbal TMs commonly used by pregnant women should not be used because of the known or potential risks associated with their use [53–55]. A multinational study conducted in Europe, North America and Australia on this issue showed that of 126 herbal medicines commonly used during pregnancy, 27 are contraindicated and 60 others require precautions [55]. It has also been reported that complementary and alternative medicines are commonly used in breastfeeding women, despite little knowledge of their safety and effectiveness during such a critical period [56].

Gastrointestinal disorders were the most expected AEs noted by the THPs concerning their products, both under the recommended conditions of use and in case of overdose, in proportions of 54% and 60.5%, respectively. This safety information derives exclusively from the empirical knowledge of THPs, based on their long experience of using their products. The population-based study in Burkina Faso noted a very frequent occurrence of AEs due to the use of TMs (14.7%) and consisting mainly of gastrointestinal disorders, at a frequency similar to that expected (57.7%) [4]. The same trend was observed in Taiwan where gastrointestinal disorders were the most frequently reported AEs of herbal TMs in the country (33.4%) [57]. Moreover, the Burkinabè pharmacovigilance system has reported that the iatrogeny of modern medicines also relates in most cases to gastrointestinal diseases (58.8%) [58]. The major role of the gastrointestinal tract in drug pharmacokinetics and the clinical symptomatology of gastrointestinal disorders would be to the disadvantage of this organ system class in drug iatrogenesis [59].

### Study limitations

Due to its cross-sectional nature, this study has certain limitations. The first is that the results of this study could not be extrapolated to the national level, due to the low participation of THPs in the study compared with the initial sample. However, given that this study analyses the phytopharmaceutical practices of THPs and that these THPs share the same socio-cultural and economic realities of the country, the results obtained might not deviate too much from the national reality.

The second limitation concerns the possible subjectivity of the declarations provided by the THPs due to a possible recall bias. To minimise this bias, the survey was conducted during the same period for all four HDs, and the questionnaires were administered to the THPs in their local languages by qualified and trained interviewers.

Despite these limitations, this pioneering study has enabled us to understand the phytotherapeutic practices of THPs and the extent to which these might impact on the conservation of plant biodiversity and the quality assurance of TMs.

## Conclusion

This study provides a panoramic view of the traditional phytopharmaceutical practices of traditional health practitioners, which is rarely explored in West Africa, and not yet been investigated in Burkina Faso. It showed that traditional practitioners, because of their medical knowledge, are an important source of health care for the population in resources limited setting. This study showed that THPs have important knowledge in the use of medicinal plants, but several shortcomings are observed in their phytopharmaceutique practices.

These findings call for continuous improvement of traditional pharmaceutical practices in order to strengthen the role of traditional medicine in promoting and protecting public health. Initiatives by stakeholders—the Ministry of Health, local authorities and THPs—should be undertaken, including the establishment of a structured programme for the cultivation of medicinal plants, training and sensitization of traditional practitioners on good agricultural and harvesting practices for medicinal plants on the one hand, and the quality and safety of traditional medicines on the other hand.

## Data Availability

The datasets used to support this study are available from the corresponding author upon request and after satisfying ethical requirements for their release.

## Abbreviations

AEs: Adverse events
HD(s): Health district(s)
THP(s): Traditional health practitioner(s)
TM: Traditional medicine
TMs: Traditional medicines
